# Author's reply

**DOI:** 10.4103/1817-1737.49420

**Published:** 2009

**Authors:** Ibrahim O. Al-Orainey

**Affiliations:** *Department of Medicine, College of Medicine, King Saud University, P. O. Box 2925, Riyadh 11426, Saudi Arabia. E-mail: iorainey@yahoo.com*

Sir,

In his letter, Dr. Al-Hajoj[1] proposes a review of BCG vaccination policy in Saudi Arabia as it is causing “more confusion than protection” and he suggests stopping BCG vaccination altogether. This despite the fact that tuberculosis incidence in Saudi Arabia is rising, according to the Ministry of Health statistics.

BCG vaccination of infants was confirmed to have significant protective efficacy against disseminated childhood tuberculosis and tuberculous meningitis. A meta-analysis of 5 randomized control trials and 8 case-control studies showed an average protection of 80% among children vaccinated in infancy.[2] The impact of vaccination was demonstrated by the decline of tuberculosis among young adults in the UK after the introduction of vaccination.[3] In Sweden discontinuation of BCG vaccination was associated with a demonstrable increase in childhood tuberculosis.[4]

Currently, BCG vaccine is not given routinely in USA, Canada and parts of western Europe, because of the low disease incidence in these countries (<5 cases/100,000). In Saudi Arabia, the incidence of the disease remains moderate (about 40 cases/100,000), and there is a high influx of foreign workers (including housemaids) from countries with high prevalence of tuberculosis. In these circumstances, exposure of children to the disease is likely to be significant; and therefore, prevention of serious childhood tuberculosis should remain a priority. This emphasizes the need to continue with the current policy of compulsory BCG vaccination of infants in Saudi Arabia. A revision of this policy is justified when the disease incidence declines to low levels.

## ERRATUM

Wali SO, Abdelaziz MM, Krayem AB, Samman YS, Shukairi AN, Mirdad SA, Albanna AS, Alghamdi HJ, Osoba AO. The presence of atypical mycobacteria in the mouthwashes of normal subjects: Role of tap water and oral hygiene. Ann Thorac Med 2008;3:5

I would greatly appreciate if you could kindly correct my academic title that appeared in the paper mentioned below which was published in Jan- March, 2008 in your Journal.

My new title is :

“The presence of atypical mycobacteria in the mouthwashes of normal subjects: Role of tap water and oral hygiene”

Correction of Primary author title:

Siraj Wali, MBBS,

Assistant professor of Medicine,

College of Medicine,

King Abdolaziz University.
